# Gate‐Assisted Programmable Molecular Doping of Epitaxial Graphene Devices

**DOI:** 10.1002/smtd.202501482

**Published:** 2025-12-03

**Authors:** Yijing Liu, DaVonne Henry, Taylor Terrones, Alexis J. Demirjian, Alexey Suslov, Valery Ortiz Jimenez, Ngoc Thanh Mai Tran, Curt A. Richter, Albert F. Rigosi, Amy Y. Liu, Nikolai G. Kalugin, Paola Barbara

**Affiliations:** ^1^ Department of Physics Georgetown University Washington DC 20057 USA; ^2^ Department of Materials and Metallurgical Engineering New Mexico Tech Socorro NM 87801 USA; ^3^ National High Magnetic Field Laboratory Tallahassee FL 32310 USA; ^4^ Physical Measurement Laboratory National Institute of Standards and Technology Gaithersburg MD 20899 USA; ^5^ Joint Quantum Institute University of Maryland College Park MD 20742 USA

**Keywords:** functionalization, graphene, molecular doping, NO_2_

## Abstract

Since the discovery of graphene, control of its carrier density via doping or functionalization has been a crucial need. Despite significant progress, precise control of the carrier density for epitaxial graphene on SiC remains a challenge. Multiple cycles of doping and characterization are often required before achieving a desired carrier density. In this work, a new approach is demonstrated to precisely program the doping level in top‐gated epitaxial graphene devices that are exposed to nitric acid vapor before the gate deposition. With the help of an applied gate voltage, the modification of carrier concentration introduced by the nitric acid can be reversibly controlled, while the corresponding carrier density at zero gate voltage can be accurately tuned by more than 4 × 10^13^ cm^−2^ across the charge neutrality point. This gate‐assisted molecular doping enables tuning of the charge neutrality point to the desired gate voltage value and can be stabilized by cooling the sample below 200 K.

## Graphical abstract

Molecular dopants encapsulated in a top‐gated epitaxial graphene device allow reversible, gate‐assisted, controlled tuning of the charge carriers by more than 4x10^13^ cm^−2^ across the charge neutrality point. This method enables device applications by compensating for the large intrinsic n‐doping of as‐grown epitaxial graphene on silicon carbide.

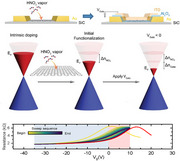

## 1 Introduction

Graphene epitaxially grown on SiC through the sublimation of Si has several advantages compared to exfoliated graphene or graphene grown by chemical vapor deposition. These advantages include scalability while maintaining high quality and no need to transfer from a growth substrate or exfoliated crystals to a device substrate.^[^ ^1^, ^2^, ^3^, ^4^, ^5^, ^6^ ^]^ Several applications of epitaxial graphene on SiC, such as terahertz detection,^[^ ^7^, ^8^ ^]^ high‐frequency transistors,^[^ ^9^, ^10^ ^]^ and quantum metrology^[^ ^6^, ^11^, ^12^, ^13^ ^]^ have already been demonstrated. Recent work also showed that it serves as a promising platform for exploring exotic quantum phenomena.^[^ ^14^, ^15^, ^16^ ^]^ However, a challenge in using this material is the intrinsic n‐doping that is due to a buffer layer formed in the sublimation process, yielding electron densities as high as 10^13^ cm^−2^.^[^ ^17^, ^18^ ^]^ Another issue is that the nonconductive SiC substrate precludes the use of a back gate, making it difficult to control the charge carrier density electrostatically.

Molecular doping of epitaxial graphene by exposure to nitric acid vapor in ambient conditions has emerged as a promising approach for varying the carrier density while maintaining high mobility.^[^ ^1^, ^2^, ^3^, ^4^, ^5^, ^6^, ^19^, ^20^, ^21^, ^22^, ^23^ ^]^ Although the process is difficult to control due to the desorption of the molecules from the graphene surface, the carrier density can be stabilized with cooling.^[^ ^3^, ^4^, ^24^ ^]^ Even so, achieving a desired carrier density requires multiple cycles of thermal annealing, dopant exposure, and magnetotransport characterization at low temperature to measure the carrier density via the Hall effect.^[^ ^1^, ^5^ ^]^ As a result, controlled change of molecular doping requires several hours and, in some cases, several days.

In graphene devices that can be back‐gated (typically with a Si/SiO_2_ substrate) the molecular doping can be tuned and monitored by measuring the shift of the charge neutrality point (CNP), eliminating the need for frequent Hall measurements. Such gate‐tunable molecular doping has been widely observed in back‐gated devices with graphene exposed to ambient conditions, where the presence of adsorbates caused hysteresis of the conductance as a function of the gate voltage. However, the change in carrier density corresponding to the shift in CNP was well below 10^12^ cm^−2^, even when sweeping the gate voltage in a wide range (± 80 V).^[^ ^25^ ^]^ Nevertheless, as mentioned above, this type of gate tuning of carrier density cannot be applied to epitaxial graphene on SiC, because it cannot be backgated.

In this work, we encapsulate molecular dopants with an aluminum oxide layer deposited on top of the epitaxial graphene. This layer also serves as a gate dielectric for our top‐gated graphene devices. We find that the charge transfer between the molecules and the graphene can be reversibly controlled by the gate voltage at room temperature, either by applying a constant gate voltage value or by sweeping it in a fixed range, allowing carrier density tunability by more than 4 × 10^13^ cm^−2^ across the CNP. This reversible control of molecular doping by applying a gate voltage contrasts with similarly functionalized, unencapsulated, and ungated epitaxial graphene devices, and it achieves wider control over the carrier density within a smaller range of gate voltages compared to graphene devices back‐gated with Si/SiO_2_.^[^ ^25^ ^]^ We show that the programmed doping level can be stabilized by cooling the samples below a threshold temperature of 230 K. The freezing of the molecular contribution to doping at low temperature allows an accurate calibration of gate voltage versus carrier density. To confirm the high quality of these devices, we perform quantum Hall measurements.

## 2 Results and Discussion

### 2.1 Sample Layout and Functionalization

Our top‐gated graphene devices are fabricated from large‐area epitaxial graphene. We first pattern Hall bars following a standard electron beam lithography process. Unless otherwise noted, the data presented in this work are collected on devices with a square geometry of side 5 µm, with two source‐drain contacts and one pair of Hall contacts. The gate dielectric is a 90 nm‐thick Al_2_O_3_ layer deposited by atomic layer deposition, and the gate electrode is a layer of 110 nm‐thick indium tin oxide (ITO) deposited by sputtering. The details of the fabrication process are outlined in the Methods section, and optical images of samples are in the (Figure S1, Supporting Information).

To compensate for the high intrinsic electron doping of the as‐grown graphene, after the Hall bar patterning, the devices are vacuum annealed at 120 °C immediately before exposure to nitric acid vapor for two minutes, following a similar process reported by Mhatre et al.^[^ ^3^, ^4^ ^]^ After exposure, they are immediately loaded into the atomic layer deposition (ALD) chamber for deposition of an Al_2_O_3_ gate dielectric.

**Figure 1:**
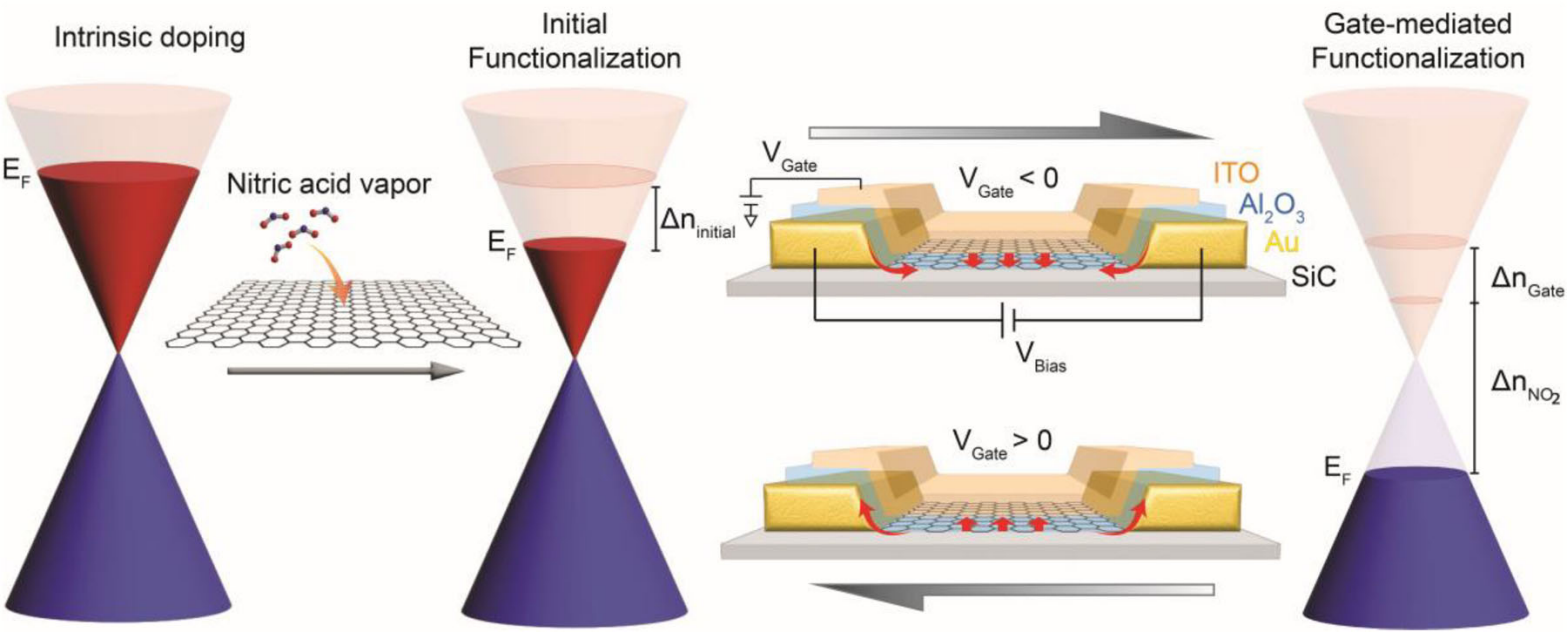
Device design and reversible molecular doping process in epitaxial graphene devices. Band diagram of graphene in a top‐gated device without exposure to nitric acid vapor, showing strong n‐doping due to the buffer layer and any other charge transfer from device processing (left). Initial functionalization by the nitric acid vapor exposure, yielding a lower Fermi energy due to p‐type molecular doping (middle). The cartoon shows the layout of devices with an indium tin oxide (ITO) top gate and an Al_2_O_3_ dielectric layer. The arrows indicate a redistribution of molecules that is reversible by changing the sign of the gate voltage. A negative gate voltage pushes additional molecules to the graphene channel, yielding additional molecular gating (Δn_NO2_), as well as electrostatic gating (Δn_Gate_), both further lowering the Fermi energy (right). A positive gate reverses the process.

In agreement with earlier reports,^[^ ^3^, ^4^ ^]^ by measuring the carrier density at different values of the gate voltage using the classical Hall effect, as described in the next section, we confirm that the nitric acid vapor p‐dopes the graphene as depicted in **Figure** **1** (middle, initial functionalization). More interestingly, upon applying a negative gate voltage, we observe an additional source of doping that augments the electrostatic gating. We attribute this additional source of doping to the redistribution of NO_2_ molecules toward the graphene surface from the metal electrode/Al_2_O_3_ interface. As indicated in Figure 1 (right, gate‐mediated functionalization), the flow of redistributed molecules is reversible upon the change of V_g_ polarity.

### 2.2 Programming the Carrier Density

Experimentally, the CNP corresponds to the resistance maximum as a function of gate voltage, where the carrier density is at a minimum and mainly determined by charge inhomogeneities, for example, electron‐hole puddles. From Hall measurements of our samples, we find that their residual carrier density is typically on the order of a few 10^10^ cm^−2^, in agreement with residual carrier density measured for epitaxial graphene on SiC in other works.^[^ ^5^ ^]^

**Figure 2:**
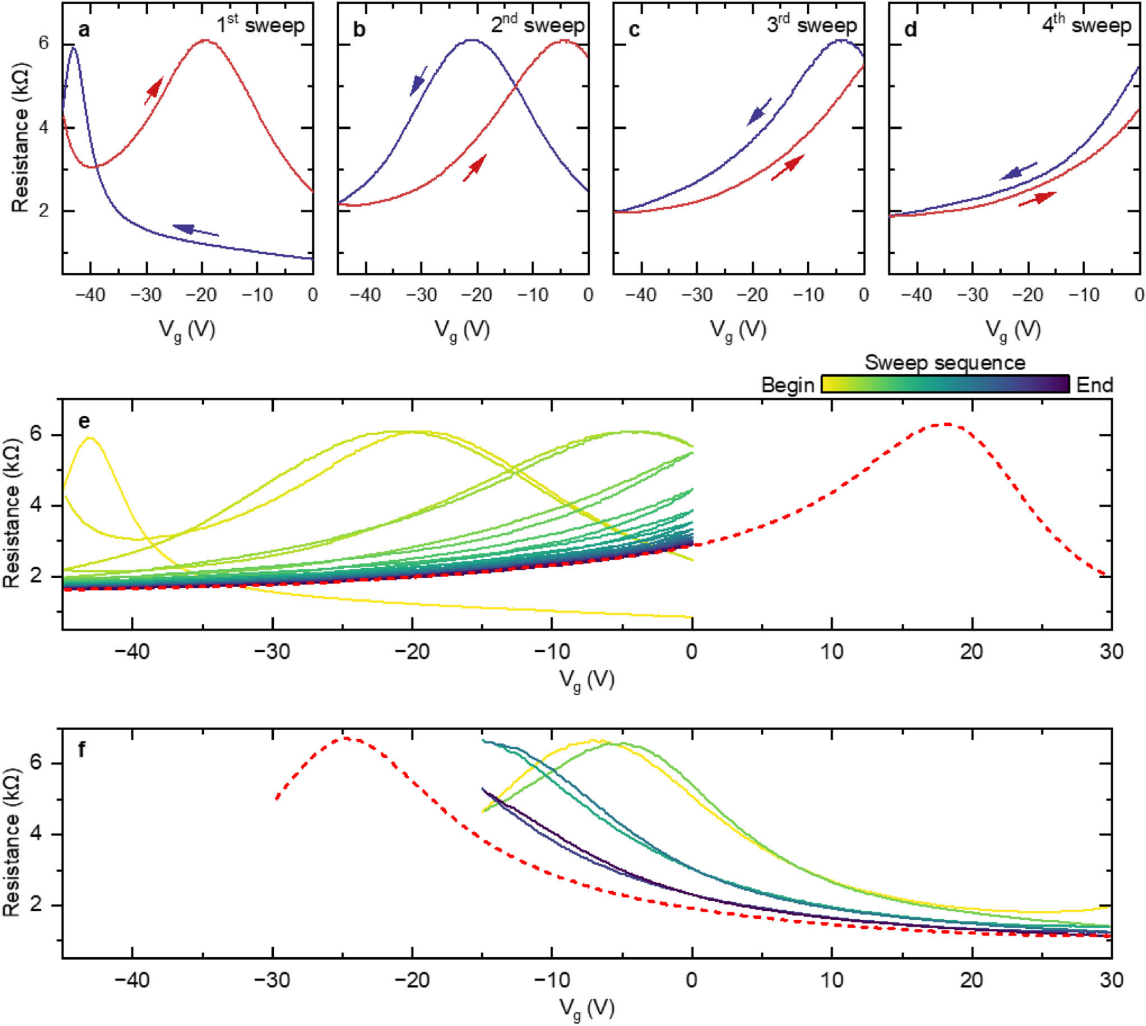
Shift of charge neutrality point during continuous V_g_ sweep at room temperature. a–d) Show the first four sweeps of the continuous V_g_ sweep, respectively. Gate sweeps in between 0 and −45 V at a rate of 20 V min^−1^. Each sweep starts and ends at 0 V. The curve colors correspond to different sweep directions as indicated by the arrows. The start point of each panel (except panel a) matches the end point of the previous panel. e,f) Continuous V_g_ sweep over different ranges (Panel a‐d corresponds to the first four loops of panel e). V_g_ sweep rate is 9 V min^−1^. In each panel, the sweep direction is no longer marked, and the color coding indicates the sweep sequence, where a lighter color corresponds to an earlier sweep and a darker color corresponds to a later one. To extract the new CNP that falls outside of the sweeping range, an extended V_g_ sweep is taken and plotted as a red dashed curve.

**Figure** **2**a shows the typical gate dependence of the electrical resistance in our devices; namely, upon sweeping toward negative V_g_ values (blue curve), we measure a classic inverted V‐shape transport curve. The negative gate voltage corresponding to the CNP indicates the graphene channel is still electron‐doped, meaning that the initial molecular doping does not fully compensate for the intrinsic n‐doping of epitaxial graphene. Since we can use Hall measurements to calibrate the correspondence between the applied gate voltage and the carrier density, our top‐gated devices provide information on the doping level from the gate voltage value of the CNP in the resistance versus gate voltage measurements, reducing the need for frequent magnetotransport measurements. In brief, for the calibration, we measure carrier density using the classical Hall effect at different values of gate voltage. From these measurements, we extract the change in carrier density Δ*n* corresponding to a gate voltage shift Δ*V*_g,_ yielding Δ*n*/Δ*V*_g_ ranging from 1.6 × 10^11^ cm^−2^ V^−1^ to 2 × 10^11^ cm^−2^ V^−1^ for different devices. An example calibration is shown in Figure S6 (Supporting Information), comparing a device with and a device without exposure to nitric acid vapor. In both cases, a linear dependence holds for carrier densities above 10^12^ cm^‑2^. At lower carrier densities, charge puddles and other traps affect this linear relationship, which fails near the CNP. This is because, in this regime, a considerable fraction of gate‐induced carriers are filling the traps rather than contributing to the electrical transport, as already discussed in previous work.^[^ ^26^, ^27^ ^]^ However, the Hall effect still provides an accurate measurement of the free charge carriers in the range where the linear relationship fails.

The exposure to nitric acid is very effective in providing an initial functionalization. When the Al_2_O_3_ is deposited as a dielectric for top‐gated devices without any exposure to nitric acid vapor, at zero gate voltage, we measure graphene electron densities higher than 1.2 × 10^13^ cm^−2^, which is similar to unencapsulated epitaxial graphene.^[^ ^17^, ^18^ ^]^ Instead, for devices with exposure to nitric acid vapor, we find that the electron density at V_g_ = 0 varies in the range 1 × 10^12^ cm^−2^ to 4 × 10^12^ cm^−2^ among different devices, confirming the initial functionalization step described in Figure 1. Yet the initial functionalization is still inefficient compared to previous reports of ungated graphene samples, where exposure to nitric acid vapor completely compensated the electron doping and even hole‐doped the sample.^[^ ^3^, ^4^ ^]^ However, as shown in Figure 2, the molecular doping in our top gated devices can be substantially increased by sweeping the applied gate voltage, even pushing the CNP well above V_g_ = 0 V. Figure 2a reveals a surprisingly large hysteresis between the sweep down to −45 V and the sweep back up to 0 V, with the CNP position shifting toward the p‐doping regime on the returning V_g_ sweep (red curve). When we continue to sweep the V_g_ for a few more cycles (Figure 2b–d), the CNP gets constantly pushed toward the positive V_g_ direction. After only four cycles, the sample becomes p‐doped at V_g_ = 0 V, and the CNP is pushed beyond the sweeping range to positive gate voltage values. With continued V_g_ sweeps, the CNP shifts further, and the curves saturate toward a repeatable trace, as shown in Figure 2e. The corresponding CNP position after saturation can be found by extending the V_g_ sweep to the positive voltage side (red dashed curve) and is determined to be at V_g_ ∼ +18 V.

Next, we sweep the gate over the same total range of 45 V with the same sweeping rate of 9 V per minute, but with V_g_ mostly on the positive side – specifically between −15 and +30 V (see Figure 2f). Such a change in the gate sweep immediately brings the sample back to the n‐doped regime, where the CNP moves back to V_g_ = −25 V after only three cycles of sweep, as indicated by the red dashed line.

The shift in the CNP described above indicates an additional source of doping that responds to an applied gate voltage. We refer to this doping source as a dynamical doping. More importantly, it appears that this dynamical doping is reversible upon changing the V_g_ polarity (see Figure 2e,f). The application of negative V_g_ results in an additional p‐type doping and vice versa. Such dynamical doping enables tuning the doping level across the CNP simply through an applied gate voltage.

**Figure 3:**
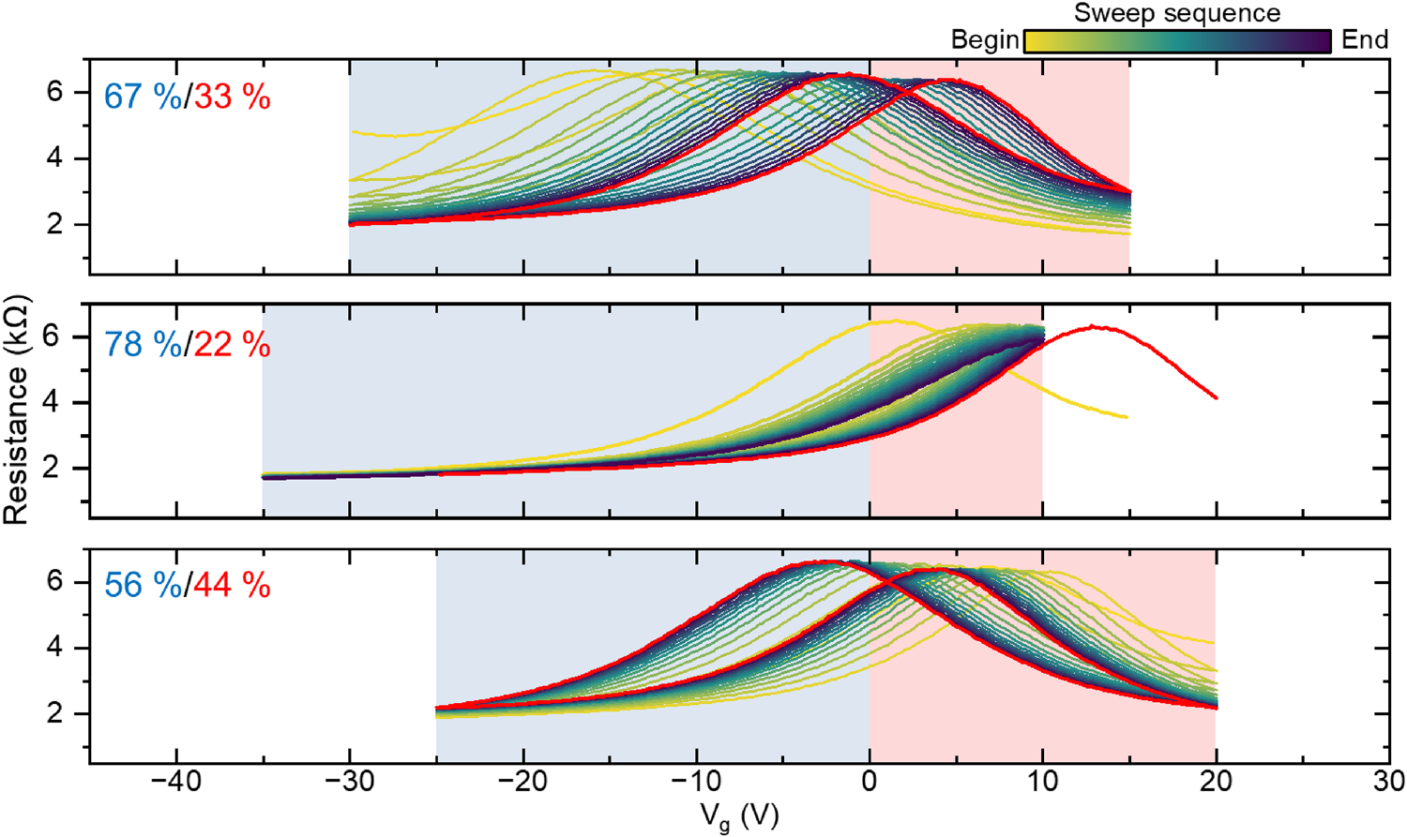
Programming of the doping level via V_g_ sweep at room temperature. Continuous V_g_ sweep over different ranges on sample A. V_g_ sweep rate is 9 V min^−1^. In each panel, the sweep direction is no longer marked, and the color code indicates the sweep sequence, where a lighter color corresponds to an earlier sweep and a darker color corresponds to a later one. In each panel, the stabilized CNP position after manipulation is determined by a V_g_ sweep plotted as a red curve. For the case where the CNP falls outside of the sweeping range, an extended V_g_ sweep is performed to extract the new CNP position. Panels are arranged by measurement sequence. The blue shade indicates a negative V_g,_ and the red indicates a positive V_g_.

Remarkably, the doping level can be easily controlled by changing the ratio of positive/negative voltage during the V_g_ sweep. If we still sweep the gate over a total range of 45 V at the same rate and vary the ratio between the positive/negative voltages during the V_g_ sweep as described in **Figure** **3**, we see that different polarity ratios result in the CNP stabilizing at different positions. For example, as shown in Figure 3a, a sweep with ‑30 V < +15 V leads to a slight p‐doping (red curve with CNP stabilizing at small positive V_g_). If one increases the weight of negative V_g_, the sample will become more p‐doped as plotted in Figure 3b. Similarly, a CNP near V_g_ = 0 can be achieved by a ratio that is close to one, as depicted in Figure 3c.

The key to tuning the dynamical doping is the presence of a gate voltage, which can be realized either through continuous V_g_ sweeps as discussed above, or by simply holding at a constant V_g_ over a certain amount of time, as discussed below. Practically, continuous V_g_ sweeps offer a specific advantage in that they enable in situ tracking of doping in the samples from the CNP position during the gate sweeps.

**Figure 4:**
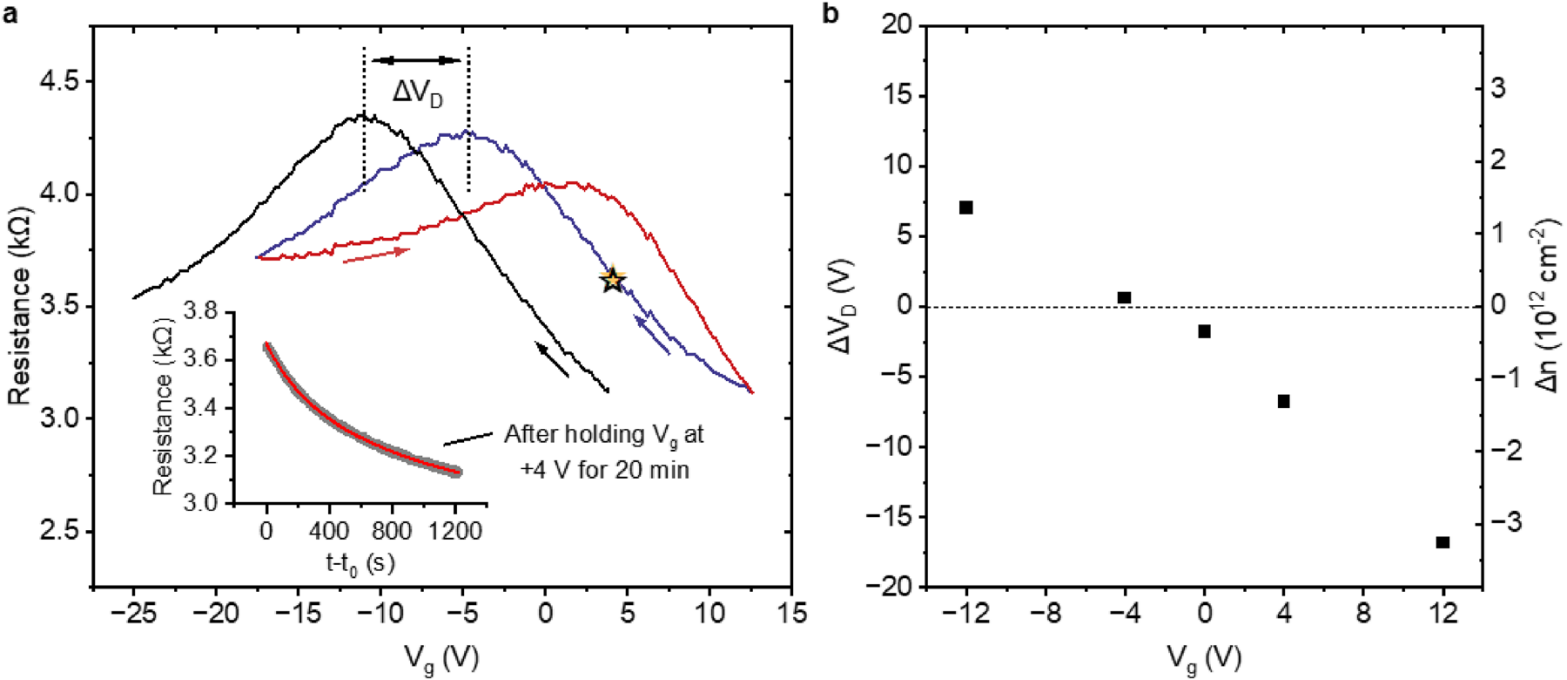
Gate‐induced shift of CNP at room temperature. a) Measurement of CNP shift after 20 min at a fixed value V_g_ = 4 V. The initial state marked with a star is obtained after sweeping the samples along the blue and red curves and then fixing the V_g_ value for 20 min, while the resistance decreases as shown in the inset. After 20 min, a downward sweep to −25 V is performed, which is plotted as the black curve. The vertical dotted line marks the CNP shift. b) CNP shifts as a function of different fixed values of V_g_ performed in the same way as the measurement shown in panel a. All measurements start from similar initial states, but the value of V_g_ that is kept fixed for 20 min is varied. If the CNP is not observed during the sweep down to −25 V, the voltage is swept up to +13 V, and then the black curve is measured on a second sweep down to −25 V. Measurements are taken on sample D.

To check the dynamical doping resulting from constant gate voltages, we measure the shift of CNP over 20 min at room temperature at different values of fixed V_g_. The results are summarized in **Figure** **4**. Before holding V_g_ at a specific value, for each measurement, we perform multiple V_g_ sweeps to prepare the sample in a state close to charge neutral at V_g_ = 0, as illustrated in Figure S4 (Supporting Information). This state is indicated by the red and blue curves in Figure 4a. The downward sweep (blue curve) is used as the reference to mark the CNP position (right vertical dotted line), and we always sweep to and pause at the target V_g_ (marked by the star) when sweeping downwards. At fixed V_g_, the sample resistance slowly decreases with time, as shown in the inset in Figure 4a. After 20 min, an immediate downward V_g_ sweep (black curve) is taken to extract the new CNP position, which is indicated by another vertical line at V_g_ = −12 V. We record the CNP shift as the interval ΔV_D_ separating the two dotted lines, and we repeat this procedure for different fixed values of V_g_. Figure 4b shows the shift of CNP ΔV_D_ as a function of each fixed V_g_ value, where we see that ΔV_D_ can be easily controlled by V_g_.

As mentioned earlier, when graphene is not encapsulated, molecular doping is often unstable due to the desorption of molecules. With the Al_2_O_3_ encapsulation, the initial functionalization (Δn_initial_ in Figure 1, that is, the molecular doping without any gate voltage applied) remains stable over ten months in ambient conditions without any gate applied (see Figure S2, Supporting Information). This is expected, since Al_2_O_3_ is known to be impermeable and commonly used as a passivation layer for NO_2_ gas sensors.^[^ ^28^ ^]^ Therefore, the concentration of adsorbed molecules is not expected to decrease due to diffusion through the gate dielectric and gate electrode.

**Figure 5:**
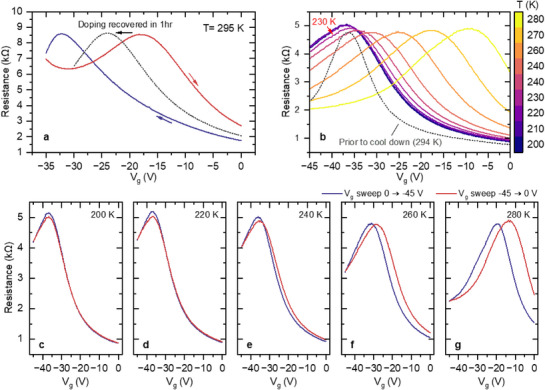
Temperature dependence and stability of the gate‐mediated molecular doping. a) V_g_ sweep (0 V → −35 V → 0 V) at room temperature for sample B. The curves are color‐coded according to the sweep direction. The slight gap between the two curves at V_g_ = −35 V is due to a ten‐second interruption in the continuous V_g_ sweep when setting up the upward sweep. The dotted curve corresponds to the V_g_ sweep taken after leaving the sample at V_g_ = 0 V for one hour, with the black arrow indicating the shift of the CNP position. b) Gate dependence at different temperatures in sample C, while warming up. The curves all correspond to the upward sweep. The dotted curve is taken at room temperature prior to cooling down. c–g) Full first loop of V_g_ sweep shown in panel b at select temperatures. The sweeping directions are color‐coded as in a.

The gate‐induced dynamical doping, although reproducible, is not stable at room temperature. When the applied gate voltage is set to V_g_ = 0, the graphene always stabilizes at an n‐doped state corresponding to the initial functionalization after waiting overnight (see blue curve in **Figure** **5**a, with a CNP gate voltage position similar to the blue curve shown for the sample in Figure 2a). For example, Figure 5a shows that the CNP is returning to the initial position (blue curve), with the corresponding electron doping increasing from n_e_ = 1.1 × 10^12^ cm^−2^ (red curve) to n_e_ = 2.4 × 10^12^ cm^−2^ (dotted curve) after one hour. Such unstable doping is not desirable for applications. However, as demonstrated in Figure 5b, it is possible to freeze the molecular doping by cooling down the device, thus enabling the stabilization of the programmed doping level, as long as the sample is kept at low temperature.

To conduct the temperature‐dependent study shown in Figure 5b, a V_g_ sweep is first taken at room temperature to determine the initial doping level in the sample (dotted curve in Figure 5b), just like Figure 5a. The sample is then kept at room temperature overnight at V_g_ = 0 V to allow the relaxation of the doping level to the equilibrium state at room temperature. Next, we cool down the sample to 200 K at V_g_ = 0 V and sweep V_g_ between 0 V and −45 V at different temperatures starting from 200 K with an increment of 10 K. At all temperatures, multiple cycles of V_g_ sweeps are used to monitor the shift of the CNP. For better clarity, for each temperature, only the forward sweeps (−45 V → 0 V) of the first V_g_ sweep cycle are plotted.

As shown in Figure 5b, the CNP position in the V_g_ sweeps at 200 K is approximately the same as it was at room temperature (dotted curve and dark purple curve, respectively), confirming the full recovery of the doping level corresponding to the initial functionalization and its freezing with the cooldown. Moreover, it is interesting to see that the resistance versus gate curves at low temperatures (200 K – 220 K) mostly overlap, while higher temperature measurements exhibit a shift in the CNP position. To take a closer look, Figure 5c–g have summarized snapshots of the first cycle of V_g_ sweep (0 V → −45 V → 0 V, the color coding refers to different sweep directions) at a few temperatures. One can see that, at both 200 K and 220 K, in contrast to the room temperature V_g_ sweeps, little hysteresis is observed, and the CNP remains stable. At higher temperatures, the CNP starts shifting measurably, although the shift is much smaller compared to that at room temperature. Similar freezing behavior in the transport curve is observed in all three additional chips we have studied, where the threshold temperature falls in the range of 230– 240 K (see Figure S3, Supporting Information).

**Figure 6:**
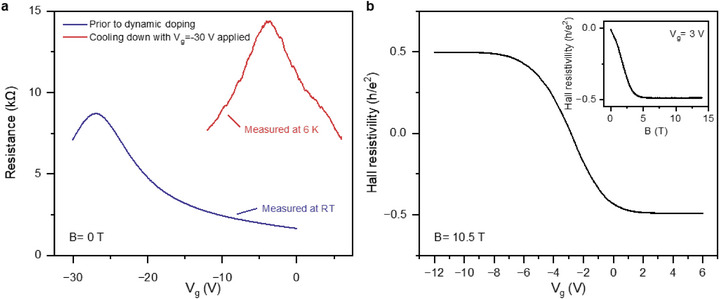
Quantum Hall plateau in the sample after tuning. a) The source‐drain resistance of sample E as a function of gate voltage at zero magnetic field before (blue curve, measured at room temperature) and after (red curve, measured at 6 K) the tuning performed by cooling down the sample at V_g_ = – 30 V. The red curve shows that the CNP is tuned close to V_g_ = 0. b) V_g_ sweep of the same sample across CNP at B = 10.5 T showing ν = ±2 plateaus. The inset shows a magnetic field sweep from 0 T to 14 T at V_g_ = 3 V with the ν = −2 plateau. Measurements are performed at T = 6 K.

A shift of the CNP can also be obtained by cooling down the sample with a constant V_g_ instead of continuous V_g_ sweeps at room temperature. For the sample in **Figure** **6**, V_g_ was first kept at −30 V for 5 min at room temperature after being swept from 0 V. The corresponding (blue) transport curve is plotted in Figure 6a, with the initial CNP at −27 V. The sample is then cooled down to 6 K at a rate of 5 K per minute with V_g_ = −30 V. The red curve shows the gate dependence after cooling down, indicating a much smaller carrier density (n_e_ = 8.3 × 10^10^ cm^−2^ at V_g_ = 0 V). The inset in Figure 6b shows the magnetic field sweep at V_g_ = 3 V, where the ν = −2 plateau starts to develop ≈5 T. Figure 6b shows a V_g_ sweep around the CNP at B = 10.5 T, and both ν = ±2 plateaus are observed. Notably, the emergence of the quantum Hall plateau confirms the high quality of the samples after gate‐tuning of their carrier density. This is very important because, although control of molecular doping in graphene is extremely desirable, for practical applications, it is also important to demonstrate that the sample quality is not degraded by such manipulation. The fact that after this controlled shift of the CNP, the samples exhibit quantum Hall effects demonstrates that the samples maintain high mobility. We note that it is difficult to estimate the mobility at room temperature for these devices due to the unstable doping above 200 K. However, assuming that the carrier density varies from 1 × 10^12^ cm^−2^ to 4 × 10^12^ cm^−2^ at V_g_ = 0, together with a source‐drain resistance ≈1 to 2 kΩ (including contact resistance). This yields a lower‐bound estimate of the mobility ≈2000 cm^2^V^−1^s^−1^.

### 2.3 Gate‐Assisted Molecular Doping: Possible Mechanism

The molecular doping originates from exposure to nitric acid vapor. In the following discussion, we focus on NO_2_ as the main constituent of the vapor because the desorption time of NO_2_ on graphene is on the order of 100 s while those of other constituents or water are much longer.^[^ ^3^ ^]^ The approximate desorption time estimated from the inset of Figure 4 is consistent with previously reported NO_2_ desorption times.^[^ ^3^, ^19^ ^]^ The capability of tuning the sample to the p‐doping regime suggests the presence of reservoirs of NO_2_ molecules that are trapped in the device away from the graphene surface. We note that although atomic layer deposition is a conformal process, which is ideal for a 3D surface such as graphene connected to source‐drain metal electrodes, the resulting Al_2_O_3_ layer is not monocrystalline, and it does not always develop with the ideal full‐coverage layer‐by‐layer growth. Submonolayer coverage per cycle leads to inhomogeneities, which can create traps at the graphene/Al_2_O_3_ interface.^[^ ^29^ ^]^ These traps may be especially prominent in regions of the device where there are sharp steps and material changes, for example, close to the graphene/electrode contact. The presence of traps and the fact that Al_2_O_3_ is a good adsorber for NO_2_^[^ ^30^ ^]^ can explain why at room temperature and without a gate voltage applied, the initial functionalization is weak: some molecules are trapped on the Al_2_O_3_ surface, away from the graphene surface. Temperature‐programmed desorption studies showed that NO_2_ can adsorb onto the Al_2_O_3_ surface and react with a surface O^2−^ ion, resulting in an adsorbed NO_3_^−^ ion. This species is bound strongly to the surface, evidenced by the fact that it does not begin to desorb until a temperature of ≈370 K.^[^ ^30^ ^]^ A negative gate voltage can encourage desorption of the charged species, resulting in the nitrate ions being desorbed from the alumina surface and converted back into the neutral NO_2_ that provides the experimentally observed p‐doping of graphene. The dipolar NO_2_ molecules can be pushed by an electric field gradient away from the traps, toward the graphene. Since adsorbed NO_2_ molecules accept electrons from graphene and become negatively charged, a positive gate voltage can encourage desorption of NO_2_ from the graphene surface. While it is experimentally challenging and beyond the scope of this work to determine the location of the traps, there is evidence that electrical contacts can play a role, consistent with the fact that, when a gate voltage is applied, the gradient of the electric field is concentrated near the interface of the graphene and gold electrodes. In samples where the thickness of contacts is reduced from 500 nm to 120 nm, yielding a flatter profile of the conducting surface formed by the graphene and the contacts, the dynamical doping is less efficient because the electric field gradient is smaller. When one repeats the room temperature V_g_ sweeps (see Figure S5, Supporting Information), the hysteresis becomes notably suppressed, and a much greater number of V_g_ sweep cycles are required before p‐doping can be achieved.

The proposed mechanism relies on a combination of the electric field gradient pushing the molecules and the field‐dependent adsorption of nitrogen oxides on alumina. The temperature below which the dynamical doping does not work is indeed very close to the NO_2_ freezing and dimerization temperature, 260 K.

**Figure 7:**
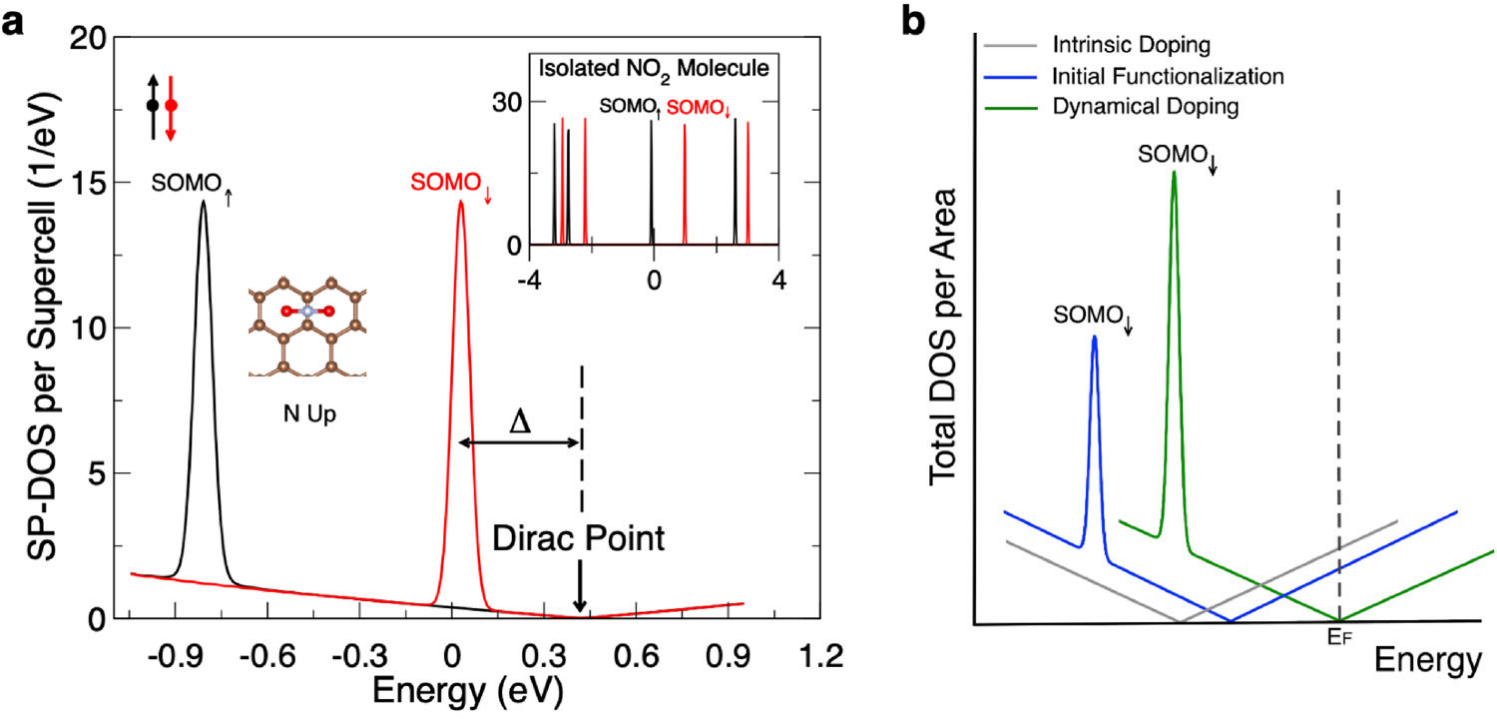
Density of States. a) DOS calculated for a 4 × 4 supercell of graphene with one NO_2_ molecule. The Fermi level for the supercell is set at zero. The inset corresponds to the DOS of an isolated NO_2_ molecule. b) Schematic of the DOS per area for the experimental protocol. Within the energy shown, there is only one molecular state shown, SOMO_↓_.

To better understand the doping effects of NO_2_ on graphene, we performed density‐functional‐theory calculations for various adsorption geometries. Consistent with prior calculations,^[^ ^31^, ^32^ ^]^ the adsorption energies have a weak dependence on orientation, as shown in Table S1 (Supporting Information) in the supplementary information. Among the geometries considered, the lowest‐energy configuration has the NO_2_ centered above a C bond, oriented with the N up (away from graphene) and the O‐O axis perpendicular to the C bond. The electronic density of states (DOS) calculated for a 4 × 4 graphene supercell with a single NO_2_ in this geometry is shown in **Figure** **7**a. Since an isolated NO_2_ molecule has an unpaired valence electron, its electronic spectrum has a semi‐occupied molecular orbital (SOMO) that is exchange split, with the unoccupied spin‐down component lying approximately 1 eV (≈1.60218 × 10^−19^ J) higher in energy than the occupied spin‐up component, as shown in the inset of Figure 7a. When NO_2_ is adsorbed on graphene, the state derived from the spin‐down SOMO (SOMO_↓_) lies ≈0.4 eV below the graphene Dirac point. While the DOS plot in Figure 7a is for a particular adsorption geometry, we have found similar results for other geometries, with Δ = *E*_SOMO↓_ – *E*_Dirac_ in the range of −0.41 to −0.25 eV.

One NO_2_ molecule per 4 × 4 graphene supercell corresponds to a coverage of 1.2 × 10^14^ molecules per cm^2^. To understand the doping efficiency of NO_2_ at significantly lower molecular coverages, we consider how the coverage affects the relative positions of the molecular states, the graphene states, and the Fermi level. With the 4 × 4 supercell, the interaction between molecules in neighboring cells is weak enough to have a negligible effect on the width of the molecular peaks and on the alignment between molecular and graphene electronic states. Therefore, at coverages at or below ≈10^14^ cm^−2^, it is reasonable to approximate Δ as fixed. The position of the Fermi level *E*_F_, however, does depend on molecular coverage. For the 4 × 4 supercell, and in the absence of other doping mechanisms beyond the NO_2_, *E*_F_ lies within the SOMO_↓_ peak, meaning that each molecule accepts a fraction of an electron from graphene. If the molecular coverage is reduced, the graphene contribution to the DOS grows relative to that of the molecule. In the limit of a single NO_2_ molecule on an infinite sheet of graphene, the SOMO_↓_ state will be fully occupied, and the infinite graphene sheet will have a single hole. In this situation, the Fermi level essentially coincides with the Dirac point. More generally, even in the presence of additional doping mechanisms such as electrostatic gating or intrinsic doping from the buffer layer, each NO_2_ molecule will accept one electron from the graphene sheet as long as *E*_F_ lies more than ≈*k*_B_ *T* above the SOMO_↓_ peak and below the next molecular peak (which is nearly 2 eV higher in energy).

To check if the electric field created by the applied gate affects the doping efficiency of NO_2_ on a per‐molecule basis, we performed calculations with an electric field perpendicular to the graphene sheet. The range of gate voltages used in our experiments is limited by dielectric breakdown effects, and the maximum applied gate voltage of |*V*_g_| ≈50 V corresponds to an average field of ≈0.5 V nm^−1^. In our calculations, for fields of *E*_z_ = ± 1 V nm^−1^, we find only small changes in the electronic DOS and Δ. For example, for the adsorption geometry considered in Figure 7a, Δ changes by about −/+ 0.02 eV from its zero‐field value. Thus, the estimated charge transfer of 1 electron to each molecule remains valid even in the presence of the electric field from the applied gate.

Based on these results, Figure 7b shows schematically the density of states at different stages of the experimental protocol. Here, the DOS is plotted on a per‐area basis, so the NO_2_ contribution grows with molecular coverage while the graphene contribution is independent of coverage. Before NO_2_ exposure, the DOS of graphene near the Dirac point is plotted as the grey curve. As grown, epitaxial graphene on SiC is strongly electron‐doped, with n ≈10^13^ m^‑2^, corresponding to the Fermi level being ≈0.3 eV above the Dirac point. After initial functionalization through exposure to NO_2_, the measured carrier concentration at *V*_g_ = 0 is on the order of 10^12^ cm^−2^ in our devices. The Dirac point shifts closer to the Fermi level (blue curve). After gate‐mediated dynamical doping that aligns the Dirac point close to the Fermi level (with carrier density < 10^11^ cm^−2^), we estimate the corresponding coverage of NO_2_ is ≈10^13^ cm^−2^, as indicated by the larger SOMO_↓_ peak in the green curve. This assumes that NO_2_ is the primary species responsible for the initial functionalization as well as for the dynamical doping. At this coverage, our model based on the DOS remains applicable.

## 3 Conclusion

Top‐gated epitaxial graphene devices provide an avenue to manipulate the carrier density of epitaxial graphene in a reproducible and controlled manner by using the gate dielectric to encapsulate dopant molecules. From our analysis, the key for carrier density manipulation is the mobility of NO_2_ dopant molecules at room temperature in response to an applied gate, with each dopant molecule acting as an electron acceptor. Remarkably, the molecule distribution on the graphene surface can be frozen below 260 K, thereby providing devices with the desired doping and stable carrier concentration that can be controlled solely by the gate at low temperature. We recently demonstrated Floquet engineering of graphene under steady‐state irradiation using this programmable doping method. Also, as shown in Figure 6 this method facilitates the use of epitaxial graphene on SiC for metrology applications of resistance standards. More generally, these findings unlock the potential of epitaxial graphene as a scalable material suitable for applications where doping control is required.

## 4 Experimental Section

### Device Fabrication

Samples were fabricated following the process developed by Yang et al.^[^ ^5^ ^]^ and adapted to electron‐beam lithography (EBL).^[^ ^14^ ^]^ The top gate dielectric was deposited by ALD using trimethylaluminum (TMA) and H_2_O as precursors and then patterned by photolithography and a wet etch step. Prior to the deposition of the top gate, devices were functionalized by exposure to nitric acid vapor for ≈2 min at room temperature, similar to previous work.^[^ ^3^ ^]^ The epitaxial graphene on SiC was purchased from Graphene Waves.

### Density Functional Theory Calculations

Density functional theory calculations were carried out with the Vienna Ab Initio Simulation Package (VASP)^[^ ^33^, ^34^ ^]^ using the projector augmented wave method^[^ ^35^, ^36^ ^]^ to treat the electron‐ion interaction and the local density approximation (LDA) for the exchange‐correlation interaction. A plane‐wave basis set with an energy cut‐off of 400 eV was used. The NO_2_‐graphene system was modeled using a 4 × 4 supercell of graphene with a single NO_2_ molecule on top. The in‐plane lattice constant was kept fixed, corresponding to a graphene lattice constant of 0.247 nm. In the out‐of‐plane direction, a vacuum layer of at least 1.25 nm was added above the molecule to minimize interactions between supercells. Geometries were optimized using an 8 × 8 × 1 grid of k‐points and a force threshold of 0.26 eV nm^−1^. A grid of 64 × 64 × 1 k‐points and a Gaussian smearing width of 0.04 eV were used for density of states calculations.

## Conflict of Interest

The authors declare no conflict of interest.

## Supporting information



Supporting Information

## Data Availability

The data that support the findings of this study are available from the corresponding author upon reasonable request.
