# Supplementary material for: Gate‐Assisted Programmable Molecular Doping of Epitaxial Graphene Devices
Source: Small Methods. 2025 Dec 3;10(3):e01482. doi: 10.1002/smtd.202501482 (PMC12893241; doi:10.1002/smtd.202501482)
Supplement: Supplementary file 1 — Supporting Information [file SMTD-10-e01482-s001.pdf]

Supporting Information

Gate-assisted programmable molecular doping of epitaxial graphene devices

*Yijing Liu, DaVonne Henry, Taylor Terrones, Alexis J. Demirjian, Alexey Suslov, Valery Ortiz Jimenez, Ngoc Thanh Mai Tran, Curt A. Richter, Albert Rigosi, Amy Y. Liu, Nikolai G. Kalugin, Paola Barbara**

Y. Liu, D. Henry, A. Demirjian, A. Liu, P. Barbara
Department of Physics, Georgetown University, Washington, DC 20057, USA
E-mail: paola.barbara@georgetown.edu

T. Terrones, N. G. Kalugin
Department of Materials and Metallurgical Engineering, New Mexico Tech, Socorro, NM 87801, USA

A. Suslov
National High Magnetic Field Laboratory, Tallahassee, FL 32310, USA

V. O. Jimenez, N. T. M. Tran, C. A. Richter, A. Rigosi
Physical Measurement Laboratory, National Institute of Standards and Technology, Gaithersburg, MD 20899, USA

N. T. M. Tran
Joint Quantum Institute, University of Maryland, College Park, MD 20742, USA

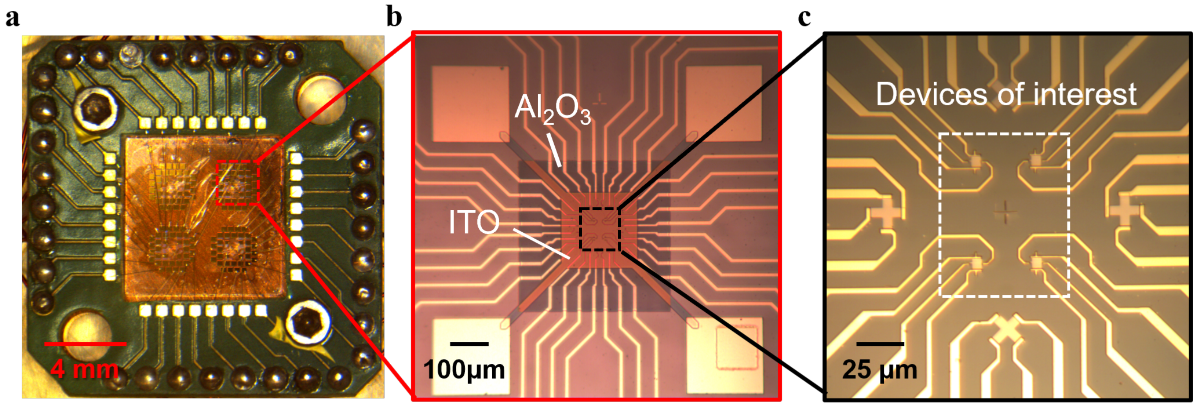

Figure S1. Top-gated epitaxial graphene FETs. a. Assembly of a typical sample with four clusters of devices mounted on a chip expander. **b**. Optical image of a cluster of four devices share a common top gate. **c**. Closer view of the devices. For a better clarity/contrast, this optical image is taken before the gate fabrication where the region with graphene still has a protective coating of 10 nm Au/Pd.

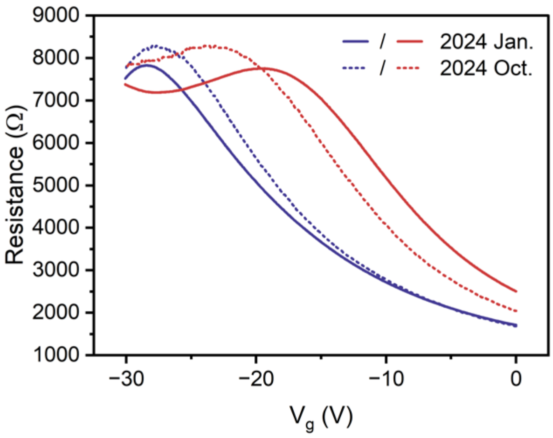

Figure S2. Stable functionalization with Al_2O_3 passivation. V_g sweeps on the same sample measured at different times. Solid lines correspond to the measurement performed in January of 2024 and dotted lines correspond to the measurement in October of the same year. For each V_g sweep loop, different colors refer to the sweep direction, whereas blue indicates the downward sweeps and red indicates the upward sweeps.

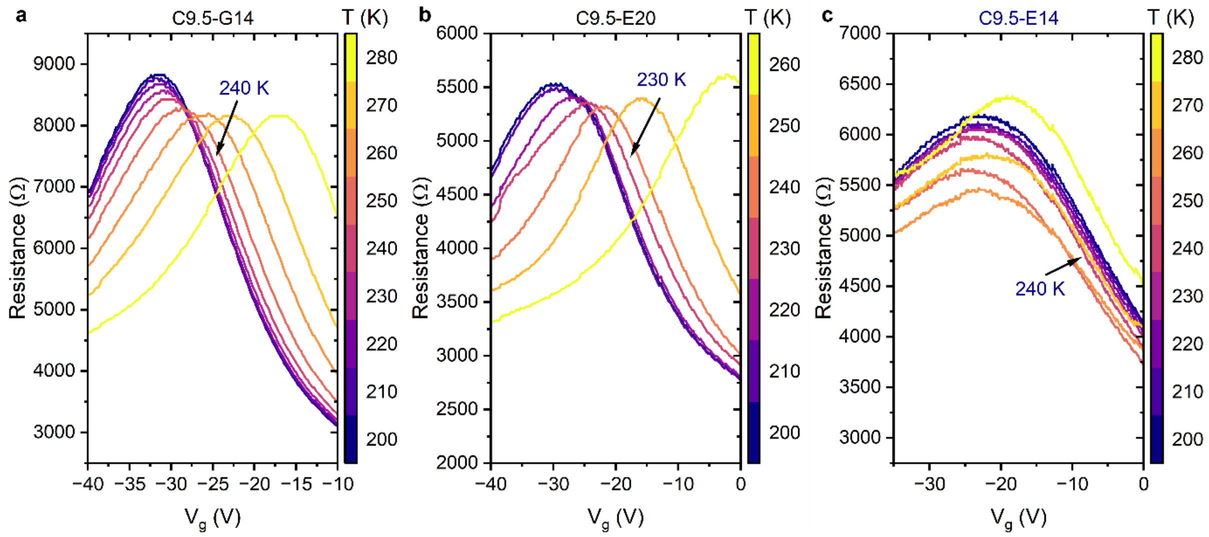

Figure S3. Temperature dependence of V_g sweeps in additional samples. Only upward sweeps are plotted for better clarity. The arrow in each panel highlights the first curve that starts to deviate and the corresponding temperature. The smaller shift in panel **c** could arise from a slower sweep rate (half of panel **a** and **b**).

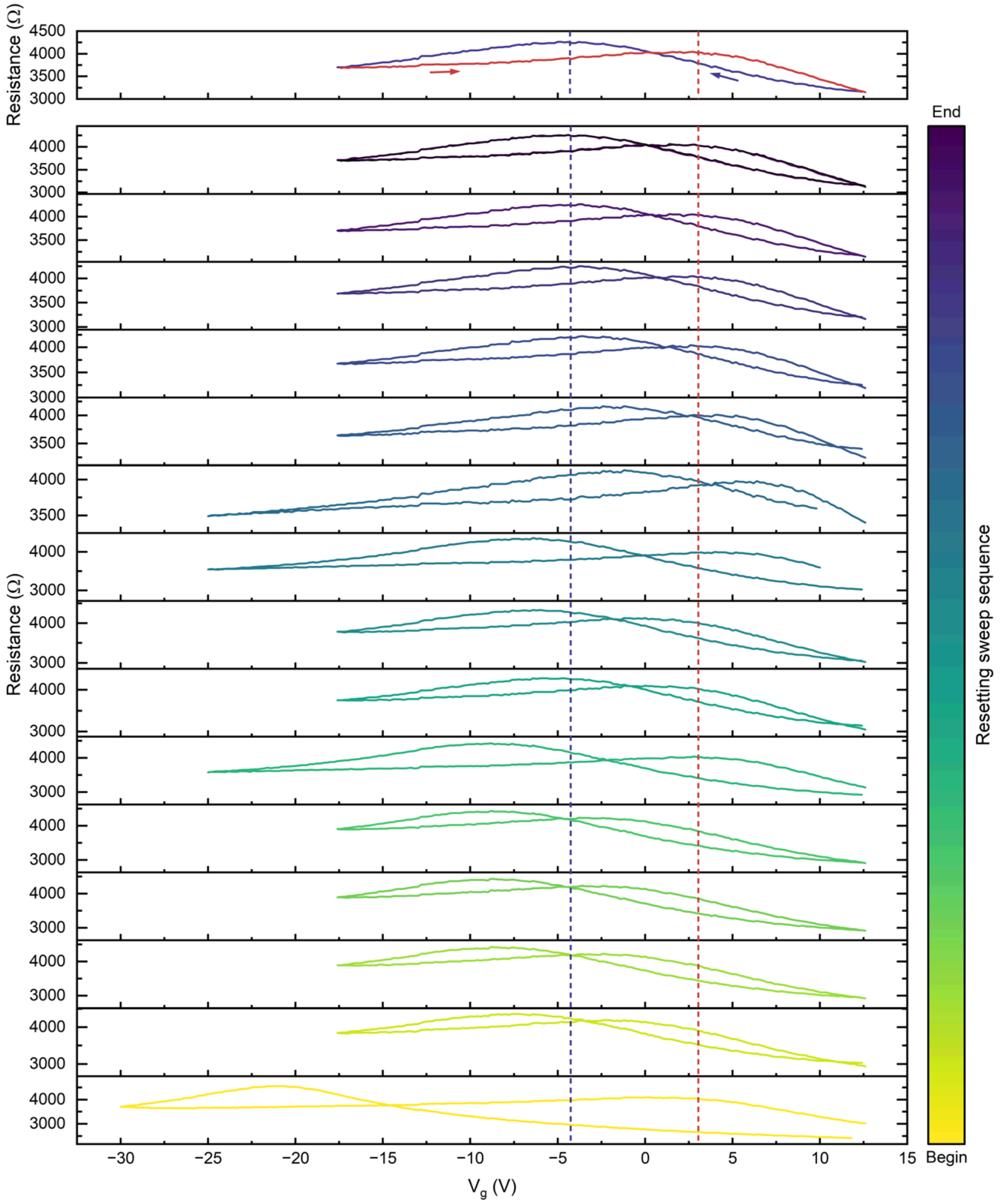

Figure S4. An example of resetting the doping level. Process to reset the doping level for the measurements presented in Figure 4. The presented data corresponds to the continuous gate sweeps to prepare the sample in the same initial state after holding V_g at 12 V for 20 minutes. The top panel corresponds to the initial state before the measurements and the bottom panel is taken afterwards. The other panels in between describe the V_g sweeps performed to reset the doping level. The color scale indicates the sweep sequence. Vertical dashed lines indicate the CNP position of the initial state. The blue and red color indicates the sweep direction, same as Figure S2.

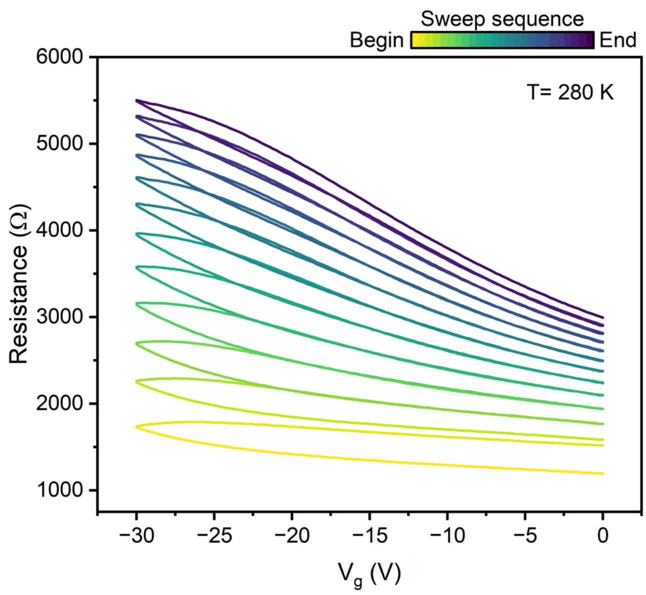

Figure S5. Continuous V_g sweep in the sample with thinner contacts. The color code indicates the sweep sequence, where a lighter color corresponds to an earlier sweep and darker color corresponds to a later one.

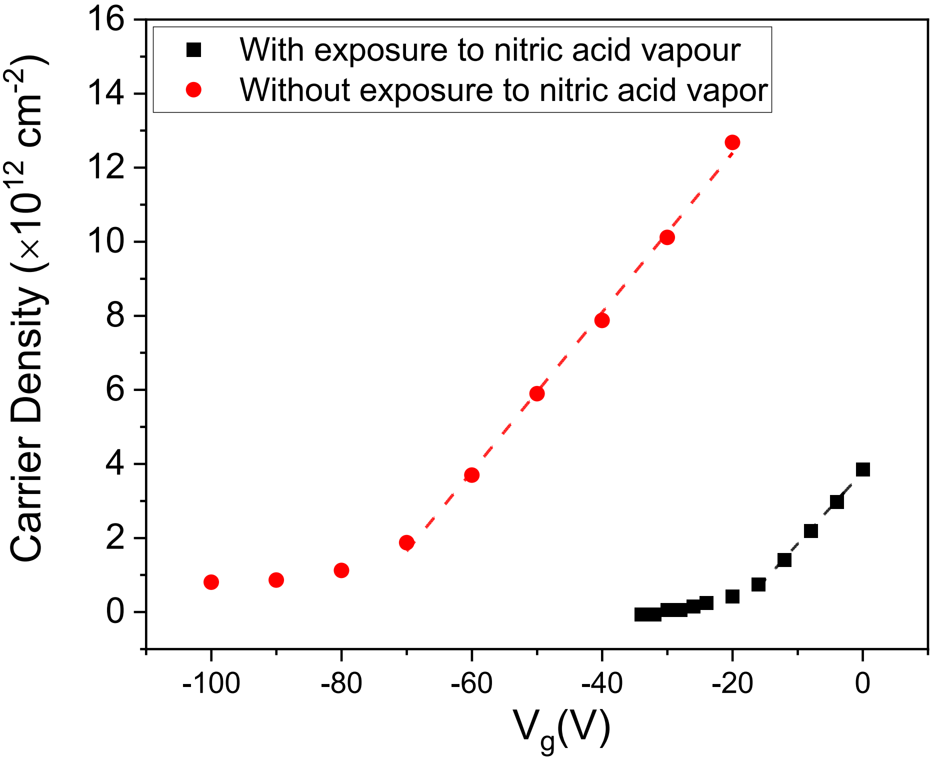

Figure S6. Carrier density vs. gate voltage calibration. The calibration is obtained by measuring the carrier density using the classical Hall effect at different gate voltages for top gated devices at low temperature where dynamical doping is frozen. The devices are fabricated with the same process, except exposure to nitric acid vapor before the gate dielectric deposition. The black data points correspond to sample B in the manuscript. The dotted lines are linear fits for data corresponding to carrier density > 1 $\times 10^{12}$cm^{-2}, with slopes 2.1$\times 10^{11}$cm^{-2}V^{-1} and 1.9 $\times 10^{11}$cm^{-2}V^{-1} for the red and black lines, respectively.

**Geometry**	**N Down [meV]** 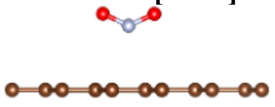	**N Up [meV]** 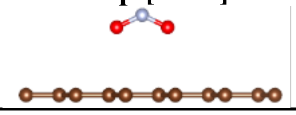
N on Top (Horizontal) 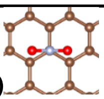	-216	-237
N on Top (Vertical) 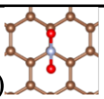	-204	-234
N in Hollow (Horizontal) 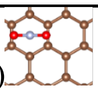	-204	-213
N in Hollow (Vertical) 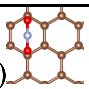	-206	-214
N on Bond (Horizontal) 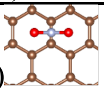	-207	-250
N on Bond (Vertical) 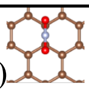	-194	-216

Table S1. Adsorption energies calculated for various geometries. Adsorption energies were computed as $E_{ads} = [E_{tot}(G + NO_2)] - [E_{tot}(NO_2) + E_{tot}(G)]$, where the same supercell was used to compute the total energy of the isolated molecule, the graphene, and the combined system. These were calculated with LDA which is known to over bind.
